# Human security at the Mediterranean borders: humanitarian discourse in the EU periphery

**DOI:** 10.1057/s41311-021-00316-1

**Published:** 2021-07-19

**Authors:** Stefania Panebianco

**Affiliations:** Department of Political and Social Sciences, University of Catania, Via V. Emanuele 49, 95131 Catania, Italy

**Keywords:** Human security, Humanitarian discourse, Mediterranean borders, EU periphery, Irregular migration

## Abstract

This article provides a re-conceptualization of human security by exploring humanitarian discourse in the EU periphery. It analyzes human security at the Mediterranean borders by focusing on humanitarian, migrant-centered discourse concerned with defending the world’s most vulnerable populations (Barnett in Annual Review of Political Science 16(1): 379-398, 2013). Empirical research has detected humanitarian discourse defending migrants’ rights, based on claims for the right to be free from inhuman treatment (Aradau in Millennium: Journal of International Studies 33(2): 251–77, 2004), as a counter-argument to the defense and closure of the borders. A humanitarian discourse focused on the alleviation of migrants’ physical and mental suffering erupted at the EU periphery when the Italian government denied a port of safety to the SeaWatch3 vessel in January 2019. This case study provides an example of center-periphery conflictual dynamics. The Italian government, defending the EU/Italian borders by closing the Italian ports, was challenged by actors mobilizing pressure, shaming the state into compliance and requesting pro-migrant legislation.

## Introduction

Security debates have traditionally characterized IR literature, as well as EU politics, and the concepts of security and borders have become closely intertwined over time. The European Union (EU) is often criticized for not being able to address security challenges at its borders, including migration, which is frequently regarded as a crucial security issue. Clearly, migration is a complex and multi-faceted phenomenon that deeply affects the borders of EU Member States (EUMS). When arrivals in Europe across the Mediterranean reached their peak in the mid-2010s, security threats permeated political discourse focusing on border closures. Center-right political leaders often depicted (irregular) migrants as criminals not to be accepted in EU territory, and adopted exclusionary policies.

Located at the center of the Mediterranean, Italy plays an essential role in securing the EU’s Mediterranean borders. In 2018–2019, the Italian government opted for a ‘closed ports’ approach, denying Non-Governmental Organizations (NGOs) conducting Search and Rescue (SAR) operations in the Mediterranean authorization to disembark rescued migrants. When, in January 2019, the SeaWatch3, a vessel of the German NGO SeaWatch, was denied a place of safety (POS) by the Italian authorities, different discourses and security priorities emerged. The SeaWatch3 blockade from 25 to 30 January 2019 in the Santa Panagia Bay, in front of the port of Siracusa, can be selected as a case study of a center-periphery cleavage where the periphery engages in humanitarian discourses, while the central government pursues an exclusionary approach to defend Italian (and hence, EU) borders.

Most of the research on EU border security has focused on securitization, conceived of as the capacity to control borders, manage threats and identify spheres of orders (Wæver 1995; Buzan et al. 1998; Huysmans 2000). The nexus between migration and security has been thoroughly explored (Huysmans and Squire 2010), and the securitization and desecuritization of migration increasingly investigated (Bello 2020). Securitization literature offers resources for understanding how policy-makers declare a condition of ‘exceptional threat’ to legitimize practices of exceptionalism. Political élites play an essential role in discursive strategy regarding the securitization of migration. Since the so-called 2015 migration crisis, migration has been openly portrayed as a security issue by European political leaders, who have often declared that their political goal is to control the borders and ‘keep people out’ (Murray and Longo 2018, 419). In the mid-2010s, migration became highly politicized in most Western European countries (Grande et al. 2019) and migration acquired salience, dominating the political debate (Hutter et al. 2016). This literature, however, neglects the humanitarian dimension of security (Barnett 2013).

A highly politicized topic like migration, which features sensitive discourses, pertains both to issues of state and human security. The politicization of migration has taken this issue out of restricted networks and into the public arena, where migration as a security issue encounters a humanitarian dimension in discourses and practices. To contrast the ‘dehumanization’ of migrants, which has been one implication of border control, humanitarian discourse focuses on migrants as individuals, in need of protection and searching for a better life. Empirical research reveals a conceptualization of human security that puts human beings, migrants in particular, at the heart of security. In the peripheries there is a well-developed humanitarian discourse defending migrants’ rights, based on claims for the right to be free from inhuman treatment (Aradau 2004), as a counter-argument to those relating to defense and closure of the borders.

This article applies the IR literature on security and borders, adopting a specific focus on human security (Kaldor et al. 2007; Kerr, 2010). It acknowledges the usefulness of existing studies on borders (‘borderlands’, in Del Sarto 2016; ‘borderscape’, in Tallis 2021), explores the tensions between humanitarianism and securitization, and investigates an underexplored research path: the center-periphery tension relating to the provision of human security at the EU’s borders. Placing the *human* at the center of EU border security is the essence of the political discourse outlined by the actors interviewed for this research, as there is ‘a humanitarian concern expressed for the lives and well-being of “irregular” migrants precisely as *humans* with the same fundamental rights as EU citizens’ (Vaughan-Williams 2015, 3). I assume that human security and the practices of humanity (Barnett 2018) can provide a comprehensive framework to understand this complex picture and to explain the political processes of securing EU borders. I adopt a ‘migrant-centered’ approach, as elaborated by several non-state actors engaged with migration management at the borders. This suggests a move to make the individual human beings the referent object of security analysis.

The article thus focuses on the humanitarian community acting at the EU’s borders, which believes in ‘emergency relief and saving lives’ of refugees and irregular migrants (Barnett 2013, 383). Humanitarian communities at the EU periphery can be very active in the provision of humanitarian practices, and actively contribute to migration governance. So far, the EU periphery has received little attention. Yet, the periphery is where humanitarian discourse develops and produces practices (Panebianco 2020a). In the EU’s Mediterranean periphery, non-state actors and humanitarian associations provide food and shelter to those in need, promote migrants’ rights and protection of the vulnerable, advocate the regularization of irregular migrants and/or the advancement of the existing legislation. These humanitarian stances can challenge central governments, as occurred in Italy with the SeaWatch3 blockade.

In early 2020, first-hand information was collected via interviews with stakeholders. Twenty in-depth interviews allowed original data on communities at the EU/Italian periphery to be collected, touching on topics such as the ‘closed ports’ policy, search and rescue of migrants in distress at sea, the externalization of sea patrolling to Libyan coast-guards, the return of irregular migrants and restrictions to international protection. This method allowed for the investigation of a humanitarian discourse that reveals a certain amount of autonomy from central government. This center-periphery gap was not dispersed by the change of government in the summer of 2019, but rather turned into humanitarian campaigns to revise existing legislation.

In several EUMS, anti-immigrant attitudes have fueled the rise of right-wing populist parties, contributing to turning migration into a challenger of the contemporary global order (Wallace Goodman and Schimmelfennig 2020). Nowadays, parties are polarized in response to issues such as migration. As suggested by Hooghe and Marks, a GAL-TAN^1^ cultural cleavage that has at its core the reaction against immigration has emerged (Hooghe and Marks 2018), and the Italian experience is meaningful in this regard. The leader of the Lega party, Matteo Salvini, has emblematically represented anti-immigrant stances and carried out what Attinà calls a ‘fencing Europe approach’ (Attinà 2018). In his capacity as former Minister of the Interior, he strengthened external borders and pursued migration control via border closure. This encouraged conflictual center-periphery dynamics and humanitarian discourses *versus* securing the borders.

This case study provides substance to the center-periphery gap concerning security at the EU’s Mediterranean borders, which is felt locally, nationally and at EU level as a matter of extreme urgency. The research questions guiding this interview-based research are the following: Are humanitarian discourses at the EU periphery dependent or independent from national security policies? Can decoupling stances develop in the periphery? The empirical analysis demonstrates that the EU periphery can operate on an inclusive logic putting human security at the center, as opposed to national cores securitizing migration. In these peripheries, networks of local authorities, civic groups and activists challenge national migration laws, policies and practices portraying decoupling attitudes.

In the following sections, I first provide a theoretical backdrop on human security at the borders. Thereafter, I explain the research questions motivating this study and the methodological choices leading to selection of the SeaWatch3 blockade as a case study. I then explore humanitarian discourse at the EU’s Mediterranean borders via empirical research, providing substance to center-periphery conflictual relations. In conclusion, I make suggestions for further research on decoupling stances in the EU periphery.

## Theoretical debates on human security at the borders

This article contributes to this Special Issue (SI) on ‘Human Security, Border (In)Security and Mobility in Security’ by focusing on human security at the borders, which implies paying attention to the security of the individual over that of the state (Huysmans and Squire 2010).^2^Elsewhere, this SI addresses the issue of the construction of a complex border space, or borderscape (Tallis 2021), where migrants experience a tough life in a sort of *limbo* of uncertainties (on the construction of ‘borderzones’, see Squire^2011^). This paper contributes to this SI, exploring human (in)security at the EU borders and non-state actors providing migrants’ security, *securing migrants* at the borders. The aim of this research is twofold: first, to challenge the existing literature on the migration-security nexus and, second, to problematize the relationship between borders and security by adding the humanitarian dimension. What matters here is the gap between the construction of migration as a national security threat and human security, qualified by humanitarian actors protecting people and providing assistance via humanitarian practices at the EU periphery.

Debates on borders are a fruitful point of departure to explore human security at the borders and move beyond the issues which have hitherto received most attention, i.e., (state) border security and securing the borders from threats according to securitization schemas. Since the late twentieth century, the concept of security, the adoption of security policies, the definition of security threats and securitization processes have been investigated under different IR theoretical approaches, and migration studies have been intertwined with security debates (Wæver et al. 1993; Buzan et al. 1998). Migration is a complex phenomenon, and a wide range of theoretical approaches and disciplines can provide a wider explanation of migratory flows. Building on research on globalization, in IR there is a wide consensus on porous borders, less on border protection, and even less on human security. Yet these are related aspects, because migration is a global transnational phenomenon touching upon migrants’ security; borders imply spaces and affect human beings across and within the borders, and the EU is no exception.

Here, attention is devoted to the EU’s external, Mediterranean borders. As Cuttitta et al. (2020, 35) remind us, ‘borders are social constructs’, and this is evident at the EU’s borders. Over time, the EU’s Mediterranean borders have been defined and redefined according to contingent needs, political interests, or specific issues at stake. Regional cooperation processes such as the Euro-Mediterranean Partnership, the European Neighborhood Policy or the Union for the Mediterranean included or excluded EU partners/neighbors, responding either to the logic of region-building or to that of bilateralism (Bicchi et al. 2018). Also in the context of migration, the EU tends to redefine the borders in a sort of border-shifting process (Panebianco 2020b).

The Mediterranean Sea, the Central Mediterranean route between Libya and Italy in particular, represents the epicenter of the migration crisis in the Mediterranean. Due to the issue of migration, Sicily, a central island in the Mediterranean, has become a key Italian/EU border. Geographers insist on Sicilian centrality in the definition of the boundaries of Europe. But Sicily is not just a transit hub, it is also a laboratory where policy initiatives and practices are elaborated and implemented. Its political role in securing the EU’s borders is crucial because Sicilian cities play essential functions in the reception of migrants and in the management of flows, securing the EU’s southern borders (Panebianco 2020a). Although politicians often draw public attention to a simplified view of migration as a security threat, appealing to the defense of national sovereignty, at the EU periphery there are actors that perform humanitarian practices, which range from urgency-practices, such as rescue at sea, first aid, providing basic provisions to the newly arrived including medical care, food, water and shelter, to longer-term interventions aimed at inclusion and eventually integration in local communities at the peripheries. By bringing in relevant human-centered features, this research re-conceptualizes the migration-security nexus in critical terms. Security represents one among other issues that impact on, shape, and constrain migration; human beings must be the central focus of the analysis.

As outposts of the EU’s external borders, the experience of Sicilian cities also draws attention to human security discourses that emerge at the peripheries and lead to ‘political borderwork’. The EU’s Mediterranean periphery is where migrants disembark and often stay due to existing legislation (or the lack thereof), although their preferred final destination would be Northern Europe. Due to the development of a restrictive EU external border regime, a political humanitarian borderwork has developed at the intersection of humanitarian intervention and border control: ‘[h]umanitarian borderwork is part of a different spatial configuration of security focused on the well-being of people on the move, while at the same time intersecting with and sometimes consolidating particular forms of territorial control enacted at the border’ (Pallister-Wilkins 2017, 6). The growing relevance of ‘humanitarian borderwork’ (Stierl 2018, 705) is becoming widely acknowledged, also due to the ever-increasing role played by NGOs in such processes. The EU’s borderwork proceeds through a fluid assemblage of functions, mechanisms, and actors; a series of loose institutional arrangements, recomposed in variable geometries ‘as necessary’ (Bialasiewicz 2012, 844).

It has to be acknowledged that, over time, technologies and practices of border surveillance have been developing at the EU’s Mediterranean borders (Oliveira Martins and Gabrielsen Jumbert 2020). When referring to the increasing use of new technologies and technical tools to control the borders, scholars talk about a ‘border industrial complex’ (Andersson 2016; Burroughs and Williams 2018; Cuttitta et al. 2020). The border security industry creates the conditions for border controls to evolve and be carried out via a range of activities. Maritime border control has exploited ‘remote control’ instruments such as offshore interdiction or detention (Zaiotti 2016), ‘smart’ border devices operating pre-emptively (Vaughan-Williams 2015) and the use of military technologies for border enforcement (Bialasiewicz 2012). In a short time, ‘a market has grown in Europe, driven by a number of firms which engage in the design, production, and supply of border security and surveillance technologies’ (Baird 2018, 119).

However, the scholarly debate on human security at the EU’s Mediterranean borders is interconnected with political considerations. Political discourse, in Italy as in other EUMS, has often insisted on closing borders as an effective political means of securing them, eventually by delegating control to third parties. When mobility is controlled beyond the territory of EUMS—with Libya as a case in point, the ‘borders of Europe’ shift to neighboring countries (Panebianco 2020b). One must distinguish between the visible *off-shoring* of European migration controls and the *out-sourcing* of migration management to African states (Bialasiewicz 2012, 848). Müller and Slominski (2020) have talked about ‘orchestration’ strategies to replace EU operations on the ground, and externalize EU migration control through the involvement of the Libyan authorities in border patrolling and SAR operations within its territorial waters. Disregarding the protection of human rights in order to escape legal constraints, orchestrators (like the EU) rely on ‘third parties acting as intermediaries’ (e.g., the Libyan government), who provide them with ideational and material support in pursuit of a certain governance goal (Müller and Slominski 2020, 1).

What renders the framework of this research more dynamic is the role of International Organizations (IOs), NGOs, Civil Society Organizations (CSOs) and volunteer groups, intervening in defense of individual migrants, thus considering individual human beings as the referent object of security (Kerr 2010). Humanitarian stances, imbued with pity and concern for the reasons that forced migrants to leave their home and/or the horrifying situations they experienced during their journey (including violence in Libya), are the main motivational factor of the humanitarian actors interviewed for this research (see Table 1). What they contest is the EU as a space of control, arguing instead for a space of secure and controlled arrival in Europe. In no case is the view of a ‘fortress Europe’ prioritizing EU border defense supported by the interviewees, who argue that states must not decide whether to accept migrants on their territory or not, because the issue of migration in the Mediterranean needs a common and stable solution based on automatic (not voluntary) mechanisms forcing EUMS to accept asylum seekers. A wide series of state and non-state actors operate at the EU’s borders, developing practices to assist migrants stuck at the borders. These humanitarian dynamics produce policies, strategies and practices to guarantee security at the borders. There is a laboratory for humanitarian discourse, either national, regional or international, focusing on migrant’s security instead of state security.

**Table 1:** Interviews on the SeaWatch3 blockade

Interview No	Institution	Function	Place of Interview	Date
I1	*AccoglieRete*	*President*	Siracusa	16 December 2019
I2	City Hall	*Mayor*	Siracusa	30 December 2019
I3	Arci	*President*	Siracusa	10 January 2020
I4	Port Authorities	*Chief commander*	Siracusa	10 January 2020
I5	Red Cross—Sicily	*President*	Catania	16 January 2020
I6	Italian Council for refugees	*Director*	Online	20 January 2020
I7	Borderline	*Spokesperson*	Catania	28 January 2020
I8	*Rete Antirazzista*	*Spokesperson*	Phone	6 April 2020
I9	*Cooperativa sociale ONLUS Prospettiva*	*President*	Catania	18 June 2020
I10	Mediterranean Hope	*Project operator*	Online	19 June 2020
I11	SeaWatch3	*crew*	Online	22 June 2020
I12	Legal Guardian		Catania	23 June 2020
I13	*Médicins sans Frontières*—Italy	*Head of Public Awareness*	Online	23 June 2020
I14	Defender of Children’s Rights—Siracusa		Phone	25 June 2020
I15	Save the Children—Italia	*Head of Unit Protection of Migrant Children*	Online	25 June 2020
I16	Save the Children—Italia	*Institutional Relationships & Advocacy Manager*	Online	25 June 2020
I17	Amnesty International—Italy	*Spokesperson*	Online	26 June 2020
I18	*Terres des Hommes*	*Advocacy Manager*	Online	26 June 2020
I19	Tribunal of Minors—Catania	*President*	Catania	29 June 2020
I20	*Associazione Diritti e Frontiere*	*Deputy*	Online	29 June 2020

## Research questions and methodological choices: assessing the Italian center-periphery cleavage on migration via a case study

This research has the theoretical ambition of sharpening the conceptual tools that are currently adopted when dealing with borders and human security, to move beyond the static analysis of linear policy-making process, and explore effective *loci* of policy-making. Once irregular migrants cross an international border, peripheral actors have specific responsibilities, performing precise tasks aimed at securing the borders. Notwithstanding this, the material situation of migrants in the peripheries depends on the legal framework set by central governments, which decide the characteristics of an ‘irregular’ migrant. Actors in the peripheries, meanwhile, play a crucial role in the management of irregular migration, being close to migrants and ‘where things happen’, where norms are ultimately implemented. The periphery reveals a certain autonomy leading to a peculiar core-periphery dynamic.

The research questions guiding this interview-based research are the following. Are humanitarian discourses at the EU periphery dependent or independent from national security policies? Can decoupling stances develop in the periphery? Empirical research suggests that there is a political center-periphery cleavage. Following the Rokkanian tradition (Rokkan et al. 1987), ‘cleavage’ in the present article refers to the deep and lasting division between actors based in the peripheries and central governments, and to conflictual discourses concerning migration. Being aware of the original party-based concept inherent to western democracies, this article suggests making the cleavage concept travel outside the founding box. A different type of political conflict has emerged. What is at stake is not the links between parties and voters, but rather different political divisions rooted in highly politicized issues such as migration. Peripheries play a crucial role in securing EU borders and represent a critical juncture in the implementation of a national legal framework that has effects both at local and EU level. Attitudes side-stepping the national government to seek more migrant-friendly regulation exist locally, alongside solidarity mobilization (Eggert and Giugni 2015; Della Porta 2018).

In January 2019, the SeaWatch3 vessel was denied a POS by the Italian authorities. This controversial state of facts resulted in a political clash between the (EU) periphery and the Italian government concerning the SAR practices due to the Italian fierce opposition to the disembarkment of rescued migrants, a position that was summed up by the so-called closed ports policy. The SeaWatch3 blockade provides concrete configuration of a center-periphery cleavage articulated around contrasting value-orientations of the groups involved (Kriesi 1998).

Migration is often regarded as threatening national security, and several European leaders have invested in populist discourses of closing the national (hence, EU) borders. The recourse to the paradigm of border security is often characterized by the language of exclusionism and national interest (Murray and Longo 2018). Exclusion versus inclusion is a political debate that has direct effects on security, and Italy is no exception. In the last few years, restrictive migration policies have been adopted by Italian governments as a reaction to this supposed threat, in the form of ‘Decreti Sicurezza’, which were introduced in 2018–2019. The Security Decrees relaunched the political debate on inclusion *versus* exclusion narratives, in particular on the inclusionary role of humanitarian NGOs and the political duty of a national government to put to an end to disembarkments on Italian soil only. A lively debate characterized the Italian political scene, with clear divisions between activists, volunteers and practitioners more inclined to a humanitarian approach in defense of migrants’ rights, and, on the other, political parties like Lega and Fratelli d’Italia totally in favor of the closure of borders.

The Lega has progressively taken an anti-immigrant, anti-refugee stance (Albertazzi et al. 2018), in line with the SAR NGOs disengagement that had already started in 2017. In Italy, the first exclusion policies were adopted under the center-left government of Paolo Gentiloni (2016–2018), when cooperation with Libya on migration became a priority to reduce the flows of arrivals. In February 2017, the Italian government signed a Memorandum of Understanding (MoU) with the Libyan government and the local forces to prevent the departures of migrants from the Libyan coasts. In July 2017, the then Minister of the Interior, Marco Minniti, introduced a Code of Conduct for SAR NGOs obliging them, among other things, to cooperate with the Libyan Coast Guard (LCG). The center-left government was threatening the closure of Italian ports to non-signatory NGOs. In June 2018 Matteo Salvini, the newly appointed Italian Minister of the Interior, declared Italian ports closed to NGOs and merchant vessels.^3^ The so-called ‘closed ports’ policy meant refusing SAR NGO vessels the permission to enter Italian ports to disembark rescued migrants. With the ‘Decreti Sicurezza’, Italian maritime and military authorities could prevent commercial and private boats that had carried out SAR in international waters from having access to Italian ports. The subsequent ‘Decreto Sicurezza bis’ allowed administrative fines against NGOs, and the seizure of vessels for NGOs that disregard the prohibition to enter Italian territorial waters. In just 5 years, the Italian government passed from having a pivotal role in SAR operations in the Mediterranean Sea, to being a frontrunner in delegating the patrolling of the Mediterranean waters to the LCG. To respond to the increasing numbers of shipwrecks in the Mediterranean, in 2013 the Italian government led by Enrico Letta adopted the *Mare Nostrum* Operation to search and rescue migrants in distress at sea. From October 2013 to September 2014, the Italian Navy conducted SAR interventions as regional practices to protect migrants, a specific category of human beings in distress, as established in International Law, patrolling the Mediterranean waters—close to Libyan national waters if necessary. In the following years, SAR operations were conducted by Italian authorities, FRONTEX and merchant vessels, with NGOs, from 2015 on, contributing to saving human lives. However, this practice became a contentious political instrument. Departing from the humanitarian essence of SAR operations, subsequent Italian governments raised the issue of defending the EU’s borders, discouraging SARs and investing instead on the externalization of EU border control via agreements with the Libyan authorities (Panebianco 2020b).

The SeaWatch3 blockade sheds light on the role that peripheries can play in fostering humanitarian discourse, irrespective of the central governments’ strategies to secure the borders. This case study unveils a core-periphery divide in the migration policy sector. Political leaders defending ‘national security’ assume a state-centric concept of security that inevitably clashes with a notion of human security centered on the insecurities of migration, including the risky conditions of human beings at sea. Humanitarian discourse condemns threats to human security, denounces migrants’ insecurity and urges instead interventions in defense of migrants to make them safe and secure.

The protection of irregular boat migrants on the high seas is a sensitive political issue. Boat migrants are subjected to an increasingly complex field of governance, in which the participating states may successfully eschew their international obligations by reference to traditional norms of sovereignty. International Law has expanded; in particular, the functioning of the maritime SAR regime has been codified in the 1974 International Convention for the Safety of Life at Sea, the 1979 Convention on Maritime SAR, and the 1982 UN Convention of the Law of the Sea. ‘Both the law of the sea and international human rights impose obligations upon states even outside their territorial waters’, however ‘rather than reducing the room for politics, paradoxically, the expansion of international law on the high seas has enhanced the possibility of sovereign and political maneuvering’ (Aalberts and Gammeltoft-Hansen 2014, 440–441).

The Italian Minister of the Interior, Matteo Salvini, for several days denied a POS to the SeaWatch3 although (or because) it had rescued 47 migrants in Libyan waters. When the vessel stopped in the Santa Panagia Bay (close to the Sicilian city of Siracusa), lawyers, activists, NGOs, but also members of the Italian Parliament ranging from rightist to leftist parties, and even the Mayor of Siracusa, protested in favor of disembarkation, considering also that among the migrants detained on board there were 13 minors. By closing the EU/Italian borders, the Italian government was implementing the ‘contained mobility’ paradigm resulting in the penalization of humanitarian SAR NGOs and the gradual operational disengagement from SAR activities by the EU and its member states (Cusumano and Gombeer 2020).

Closing the EU/Italian borders has an enormous political impact, with IOs, NGOs and local actors (religious groups, activist networks, volunteers) raising the issue of deaths at the borders.^4^ The political rhetoric of border closure was increasingly presented by the Italian government as being aimed at preventing deaths, and thereby justified. Protecting the lives of migrants has long been one of the declared aims of restrictive border policies. Institutional efforts aimed at fighting human smuggling and preventing migrants from embarking on irregular journeys to Europe has become a recurrent narrative of EUMS investing in the closure of borders to provide and guarantee border security. Borders are sites of performing sovereignty (FitzGerald 2020), where control over mobility can turn into border protection and even border closure. Border control, often assumed as the essence of state-sovereignty, can clash with the protection of irregular migrants. To face the Mediterranean migration crisis erupted in 2015, several EUMS adopted restrictive measures and tended to justify barriers to migration to defend national security concerns.

However, qualitative data collected via interviews assessed the existence of a humanitarian discourse at the EU periphery as a counterweight to center-led border control. The content and meaning of statements specified in Table 2 concerning border protection via borders (ports) closure, including legal aspects and interpretations that have entered the Italian political debate and attracted the public attention, were tested with relevant stakeholders. They regard migrants, children in particular, as the prime beneficiaries of local policy measures due to their vulnerability, their basic needs for shelter, health care, education, etc. Data collection was conducted in late 2019–mid-2020. Twenty in-depth interviews were conducted in Siracusa, Catania and online, to investigate on the SeaWatch3 blockade. All interviewees listed in Table 1 had an active role in the blockade of the SeaWatch3. Port Authorities, NGOs, religious groups, activists, volunteers, the City Mayor of Siracusa, a magistrate, a legal guardian and the SeaWatch3 crew were addressed via semi-structured interviews. The author’s pre-existing contacts were exploited to refocus stakeholders, and a snow-ball method allowed for the reconstruction of the spontaneous protest against the decision of the Italian government forbidding disembarkment for the migrants rescued by the SeaWatch3.

**Table 2:** Do you agree/disagree with the following statements?

Statement	I fully agree	I agree	Don’t agree, nor disagree	I do not agree	I fully disagree
Any EUMS must decide whether to accept migrants on its territory or not			X	XXXXX X	XXXXX XXXXX XXX
State security prevails over human security of migrants entering the EU irregularly			X	XXXX	XXXXX XXXXX XXXXX
The issue of migration in the Mediterranean needs a common and stable solution based upon automatic (not voluntary) mechanisms forcing EUMS to accept asylum seekers	XXXXX XXXXX	XXXXX XXX	X	X	
When necessary, human rights promotion and vulnerable protection must imply activities of human rights promotion and protection of vulnerable people moving beyond existing norms	XXXXX XXXXX XXXX	XXX			XXX

## Humanitarian discourse in the periphery challenging Italian borders’ closure: the SeaWatch3 blockade

The ‘closed ports’ policy declared by the Italian Ministry of the Interior in June 2018,^5^ and the ensuing refusal to let SAR NGOs’ vessels enter Italian ports, marked a peak in tense relations between the Italian government and humanitarian actors broadly involved in migration management at the peripheries. The questions whether or not private actors can exert the legal duty to render assistance to migrants and others lost at sea, and whether coastal states such as Italy must disembark those rescued at a POS, entered the public debate. SAR operations have become a very divisive political issue both at EU and national level and the SeaWatch3 blockade off the Siracusa coasts in January 2019 represents a case in point.

Claims related to migration have entered the political arena and ‘avoiding excessive immigration’ has become one of the most relevant conflictual issues in Europe (Hutter et al. 2016, 186). As Hooghe and Marks (2018) argue, migration has become a political issue that polarizes political actors and public opinion around a GAL-TAN cleavage, where identities and cultural belonging are the core of the political competition. This new divide does not build around traditional party political competition, but rather around migration, a highly politicized transnational issue. For parties from the radical populist right, opposing irregular migration tends to be a central position. Not surprisingly, none of the interviewees listed in Table 1 located themselves at the right hand extreme of an ideal left–right continuum. Nonetheless, not all would locate themselves at the left hand extreme.

Over time, individual behaviors in the peripheries have produced collective behaviors that in the case of the SeaWatch3 produced protests against the decision of the Minister of the Interior not to assign a POS. Migration is inherently related to protest categories, in both senses of protests against or in favor of irregular migrants. But this was not a protest, an occasional mobilization, it was rather an example of collective voice, that structured and channeled individual voices and expressed the political stances of a vibrant peripheral community. Considering that most of these actors have been in the field for more than 15 years, one of the two poles of the GAL-TAN cleavage has become rather consolidated. This cleavage challenges the primacy of the central government in framing discourses on migration in terms of mid- to long- duration. Empirical investigation indicates that the periphery can show a territorial distinctiveness; in the EU periphery there is a consolidated humanitarian discourse shared by large sectors of society, dealing with migration management and putting human beings at the center. By their actions, therefore, they oppose a political narrative centered on exclusionary discourse.

Humanitarian discourse in the EU periphery exists, challenging and contesting the Italian government’s exclusion policies. Considering that SAR operations are a fundamental humanitarian principle, a pillar of the regime at sea and a crucial norm of international law, the widespread opinion was that the SeaWatch3 should be assigned a POS to disembark rescued migrants. SAR NGOs, in fact, have ‘humanitarian reasons’ to apply and ‘fill the gap left by state actors’ (I11). Alongside the SeaWatch crew (I11), various actors expressed fierce criticism of the decision of the Italian government. The Mayor of Siracusa (I2), several NGOs and CSOs contested the concept of ‘fortress Europe’ that the Italian government was pursuing with its ‘closed ports’ strategy. Arci (I3), the Italian Council for Refugees (I6), Mediterranean Hope (I10), *Médecins Sans Frontières* (I13), Save the Children (I15, I16) and Amnesty International (I17), among others, signed a letter to the Prime Minister Giuseppe Conte, urging him to let the minors disembark (see Table 3). *Terres des Hommes* (I18) also addressed the President of the Italian Republic, while Rete Antirazzista (I8), Legal Guardians (I12), the Defender of Children’s Rights (I14) and lawyers (I20) reacted to the deprivation of liberty of the irregular migrants on board, who had not been identified as potential asylum-seekers, and promptly set up grassroots mobilization to co-ordinate activists’ demands for human rights and legal protection. This was not just regarded as a humanitarian emergency, but rather as a severe violation of human rights, especially minors’ rights, as the President of the Tribunal of Minors in Catania declared (I19).

**Table 3:** From Humanitarian Discourse to Action: outputs

Institution	Petitions	Mobilization campaigns	Advice for legislation
*Amnesty International* *Arci* *Médecins sans Frontières* *Mediterranean Hope* *Save the Children* *Terres des Hommes*	Letter to the Prime Minister requesting the disembarkation of migrants rescued by SeaWatch3, January 2019		
*Rete Antirazzista* *Cooperativa Prospettiva*		LasciateciEntrare	
*Accoglierete* *Save the Children* *Terres des Hommes* *Tribunal of Minors Catania*			Legge Zampa 47/2017

Actors ranging from IOs to NGOs, CSOs, activists and volunteers protested against the decision of the Italian government not to provide the SeaWatch3 a POS. A well-organized network providing first aid, shelter and assistance to those in need, irrespective of their *irregular* immigration status, exists in the Siracusa area. The local community is very active in providing services to migrants, to remedy inhumane and unsafe life-conditions. Humanitarian practices have developed over time in a learning-by-doing process, which is favored by experts in the migration field (Panebianco 2020a). There is a vibrant community of humanitarian actors who do not act as mere agents in a principle-agent framework where the national authorities adopt norms that they merely accept, passively implement and comply with. Different discourses, interests and views exist in the EU periphery leading to specific action which considers first and foremost the migrants’ needs, which may be conducive to free-riding or even decoupling. The SeaWatch3 blockade caused protests and demonstrations against the central government and the ‘closed ports’ approach, summed up in tweets by Minister Salvini: “… having stayed for days in Maltese waters, SeaWatch3 with 47 on board is heading for our shores. No one will disembark in Italy” and “[r]eady to send medicine, food and whatever is necessary, but Italian ports are and will remain closed” (on 24 January 2019).

In the days of the blockade a clear humanitarian discourse emerged, which requested not just the disembarkment of rescued migrants but also the reintroduction of the suspended international protection and the regularization of irregular migrants (I3, I6, I8, I9, I18, I20). These actors, with the Mayor the most prominent of all, challenged the Minister of the Interior and the ‘closed ports’ policy approach, by requesting the revision of a highly controversial existing national legal framework.

There is a lively scholarly debate on EU externalization to neighboring countries such as Turkey, with the EU-Turkey statement of 2016, or Libya, with the agreement of January 2017, without obtaining reassurances about humane treatment (Mourad and Norman 2020). Humanitarian associations and international human rights groups also denounce the human costs of these agreements (I10, I12, I14, I17, I18, I20). Human security is endangered if the EU borders are secured via agreements with third countries that do not respect human rights, when migrants are subject to inhumane conditions. This has raised concerns about cooperation with the Libyan authorities by involving the LCG in sea patrolling, considering their low respect for international law and lack of minimal standards of human rights.^6^ IOs such as the International Red Cross (I5), and NGOs such as Amnesty International (I17), have denounced casualties at sea, but also abuses perpetrated by smugglers and the LCG. With their critical opinions on the Libyan role in managing migration, they upgraded this domestic debate into an international one.

Most interviewees claim that Libyan methods are disrespectful of human rights, both during SAR operations and in the hosting centers on the homeland. Hence, they argue, when dinghies are at risk in Libyan territorial waters, a humanitarian logic must prevail and involve SAR operations, also considering that under International Law the duty to render assistance always prevails. International maritime law imposes an obligation to guarantee swift disembarkation to people in distress at sea in a POS, especially when minors are at risk (I19). However, the definition of ‘place of safety’ remains contested.^7^ Legal associations recall that the European Court of Human Rights, in its landmark 2012 Hirsi case, confirmed the extraterritorial reach of the human rights protection regime when assessing Italian authorities ‘pushbacks’ to Libya of people intercepted at sea (I20). The UNHCR (UN Refugee Agency) has repeatedly declared that Libya is not a viable POS and the International Organization for Migration (IOM) argues that states should avoid actions (or inactions) leading to disembarkation in unsafe territories such as Libya, where people are at risk of torture or other inhuman or degrading treatment. Moreover, Italy has been fiercely criticized for providing patrol boats and training the LGC, thus empowering the LCG through its direct assistance and international endorsement (I20).

Fieldwork allowed us to assess that the discourse of securing the borders by closing frontiers is fiercely rejected by those actors who manage migration at the EU periphery, or conduct advocacy at EU and national level to revise legislation to protect migrants’ rights (I6, I17, I20). For most interviewees involved in humanitarian action, securing migrants at the borders implies first and foremost ‘not dying while crossing the borders’ (I11). There is a well-grounded network of NGOs, CSOs and activists transforming discourse into concrete action. The legal campaigns seeking the revision of existing norms (the ‘Decreti Sicurezza’ in particular) deserve a special mention (Table 3).

This spontaneous political and legal movement in favor of the disembarkation of rescued migrants seems in line with the role that religious associations usually play in helping those in need, migrants included (I10). Yet this is a much broader process, giving substance to a humanitarian discourse centered on migrants’ rights, and arguing for the revision of inadequate existing legislation or the filling of a normative vacuum. The stakeholders’ expertise is well-known and can contribute to the elaboration of legislation, as in the case of the ‘Legge Zampa 47/2017’ for minors’ protection, that profited from the knowledge of the Tribunal for Minors in Catania (I19).

The analysis of the SeaWatch3 blockade has revealed a confrontational center-periphery attitude that clearly emerged when the municipal authority expressed a different political stance compared to that of the central government. When interviewed, the Siracusa Mayor Francesco Italia expressed his ideological commitment to the migration cause and explained his pro-migrant behavior and conflictual stance during the SeaWatch3 blockade as ‘natural’ and ‘obvious’ conduct (I2). He expressed himself in favor of the migrants’ disembarkment via media and speeches, yet the actual decision was in the hands of the Minister of the Interior. This created legal uncertainties and increased the migrants’ hopes and expectations (I11) until the Minister of the Interior had to authorize, 6 days later, their disembarkment. Minors were disembarked first, primarily thanks to the legal action of the Guardian of Children’s Rights in Siracusa (I14) and the application of existing norms of child protection orchestrated by the Tribunal of Minors of Catania (I19) which had appointed legal guardians for them (I12).

## Conclusion

Departing from studies on securitization of migration, this article has shed light on migrants’ insecurity at the EU’s borders and explored the center-periphery gap that can emerge in discourses on securing migrants at the borders. In particular, it touched on territorial borders and the essential boundary function of peripheral EU areas, where humanitarian discourse and solidarity practices have emerged over the years. Actors in the EU periphery play a crucial role in elaborating a humanitarian discourse that combines narratives and practices and can produce distinct results from the national cores. The SeaWatch3 blockade in January 2019 demonstrated that, at the EU periphery, there is a humanitarian community that attempts to save lives, provide emergency aid and legal counseling to reduce the suffering of vulnerable groups such as irregular migrants.

The EU periphery hosts a wide range of humanitarian non-state actors, IOs, NGOs, CSOs, activists, religious groups, etc., experiencing humanitarian practices to provide migrants’ security and expressing humanitarian discourse even if this goes against the prevailing governmental discourse. This article contributes to the developing literature on humanitarian interventions conducive to an international humanitarian order that provides assistance to vulnerable subjects. The empirical research underneath demonstrates the existence of a humanitarian community articulating humanitarian discourse; those interviewed in this research are committed to intervene in favor of irregular migrants, they are willing to ‘make noise to bring attention and assistance to them’ (Barnett 2013, 380). Interviews were consistent on this point: irregular migrants are regarded as vulnerables deserving protection, as they are victims of poverty, often in the hands of smugglers and subject to atrocities during their journey. Non-state actors are committed to securing borders, and do not dare to express their voices to reduce migrants’ suffering, proposing solutions which might include decoupling behaviors.

The SeaWatch3 blockade indicates that peripheries can be more pragmatic, reactive and receptive to migrants than state actors, but also proves that humanitarian stances develop ‘where the problem arises’ (Spencer and Delvino 2019). The article has contributed to unpacking the political dimension of migration by focusing on the local actors that provide substance to migration policies and play a crucial role in the EU-EUMS game. The EU has no real influence to secure the borders, while EUMS are essential, together with those actors operating on the ground. Decisions adopted at EU/EUMS level are implemented through local actors, or international actors acting locally.

In January 2019, when the vessel of the German NGO SeaWatch was denied entry into the port of Siracusa, its Mayor announced the intention to disembark rescued migrants. Siracusa is a key site on the EU borders, where irregular migrants often remain stuck. Over the years, peripheral cities such as Siracusa have provided humanitarian assistance to irregular migrants (Panebianco 2020a), while national exclusionary measures were implemented against unauthorized migration. Municipal administrations and civic institutions, together with religious groups, as happens in other cities in the world, have created an environment of hospitality for migrants and refugee (Bauder 2017). This empirical research on mobilization in favor of migrants’ disembarkation from SeaWatch3 has traced the existence of a pro-migrant movement that is embedded in the EU periphery.

The impact on human security of free movement, of free entry or, conversely, of closed borders and control at borders was the core of this empirical analysis. Humanitarian stances are those taken by humanitarian associations and various actors in the peripheries, who help irregular migrants, asylum-seekers and vulnerable subjects such as unaccompanied minors or trafficked women. When border security is at stake, a conflictual relation can emerge, bringing divergent discourses and decoupling stances and policies to the fore. Decoupling attitudes exist alongside solidarity mobilization. The EU periphery reveals a general commitment to welcoming asylum seekers and refugees. In an extreme sense, humanitarian discourse can challenge national sovereignty, as the SeaWatch3 blockade demonstrated. Further investigation is needed to verify which particular national circumstances enable or constrain various aspects of decoupling attitudes.

## Footnotes

1 GAL refers to the ‘green/alternative/libertarian’ pole of a continuum with TAN (‘tradition/authority/national’) at the opposite pole.

2 In early 2020, the Covid19 pandemic has relaunched the political debate on state borders’ closure *versus* regular(izing) opening.

3 The arrival of large number of irregular migrants entails logistical challenges and specific operational tasks, therefore disembarkation can only occur where hotspots for migrants’ identification are available. De facto*,* the Ministry of the Interior enjoys a veto power over the disembarkation of migrants on the Italian territory.

4 According to the International Organization for Migration, from 2014 to 2018, an estimated 15,062 people died while crossing the Central Mediterranean route, making it the deadliest migration route in the world. IOM Missing Migrants Project data indicate that the rate of deaths per number of people attempting the journey increases. The rate went from one death for every 38 arrivals in 2017 to one for every 14 arrivals in 2018 (https://missingmigrants.iom.int/).

5 The closure of the Italian ports was first experienced in June 2018, when the Aquarius ship, operated by *Médicins sans Frontières* and the German NGO *SOS Méditerranée*, was denied access to Italy’s territorial waters. The Spanish government then decided to allow disembarkation of the migrants in the port of Valencia.

6 Figures of people rescued/intercepted at sea by the LCG show that, in 2019, the LCG rescued/intercepted a total of 9,035 people in different locations along the Libyan coast (data accessed on 25 April 2020). https://data2.unhcr.org/en/documents/download/73284.

7 The Decree of the Italian Court in charge of Ministers’ crimes stated that such an ‘obligation of conduct’ cannot represent an obligation to disembark irregular migrants rescued by SAR NGOs and, as in the SeaWatch3 blockade, states retain the right to allow or deny access to their national ports (Court of Catania, Section Ministerial Crimes, 18/6/2019).
